# Definitions of determinants of physical activity behaviour: process and outcome of consensus from the DE-PASS expert group

**DOI:** 10.1186/s12966-025-01728-5

**Published:** 2025-03-18

**Authors:** Jan Dygrýn, Javier Brazo-Sayavera, Joana Cruz, Mekdes Kebede Gebremariam, José Carlos Ribeiro, Laura Capranica, Ciaran MacDonncha, Yael Netz

**Affiliations:** 1 Faculty of Physical Culture, Palacký University Olomouc, třída Míru 117, Olomouc, 779 00 Olomouc, Czech Republic; 2 Department of Sports and Computer Science, Universidad Pablo de Olavide, Seville, Spain; 3 Center for Innovative Care and Health Technology (ciTechCare), School of Health Sciences of the Polytechnic University of Leiria, Leiria, Portugal; 4 Department of Community Medicine and Global Health, Institute of Health and Society, University of Oslo, Oslo, Norway; 5 Sustainable Health Unit, University of Oslo, Oslo, Norway; 6 Research Centre in Physical Activity, Health and Leisure, Faculty of Sport, University of Porto, Porto, Portugal; 7 Laboratory for Integrative and Translational Research in Population Health, Porto, Portugal; 8 Department of Movement, Human and Health Sciences, University of Rome Foro Italico, Rome, Italy; 9 Department of Physical Education and Sport Sciences and Health Research Institute, University of Limerick, Limerick, Ireland; 10 Levinsky-Wingate Academic College, Wingate Campus, Netanya, Israel; 11 Department of Health Promotion and Rehabilitation, Lithuanian Sports University, Kaunas, Lithuania

**Keywords:** Movement behaviour, Active lifestyle, Health, Settings, Delphi method, Consensus

## Abstract

**Background:**

Despite extensive research on physical activity behaviour (PAB), consensus is lacking on related terms and definitions, thereby hindering the ability to compare findings between studies and to develop reliable assessment tools. This study therefore aimed to establish consensus on the definitions of key PAB determinants.

**Methods:**

First, an international expert steering committee was established, comprising members of the European Cooperation in Science and Technology (COST) action “DEterminants of Physical ActivitieS in Settings” (DE-PASS). Recently published review-level studies were used to identify key determinants of PAB. Two independent reviewers systematically reviewed the literature to catalogue the range of definitions used for key determinants of PAB (steps 1–2). A two-round modified Delphi survey was conducted online from February to September 2023, to determine the optimal definition for each determinant. In round 1, experts selected the most suitable definition for each of the 41 initially identified determinants. In round 2, experts ranked the appropriateness of the definition selected from round 1 on a 5-point Likert scale. Consensus was defined a priori as ≥ 75% agreement on the definition (i.e., ratings of ≥ 4 points). A professional English language expert ensured concise, coherent wording and high-quality editing of the definitions (steps 3–6).

**Results:**

Eighty-five experts in PAB research participated in round 1, and sixty-nine experts in round 2. Consensus of definitions was achieved for 39 of the 41 determinants (88.4%–98.6% agreement). The consensus threshold was not achieved for two determinants: genetic profile and regulation (69.6%) and backyard access/size (73.9%).

**Conclusions:**

The findings of this study offer a consensus-based set of definitions for 39 key determinants of PAB. These definitions can be used homogenously in academic research on physical activity.

**Supplementary Information:**

The online version contains supplementary material available at 10.1186/s12966-025-01728-5.

## Background

Paradoxically, while overwhelming evidence highlights the health benefits that are associated with optimal physical activity [1–3], global trends show significant reductions in physical activity. These declines, coupled with an increase in excessive sedentary behaviours [4–7], have prompted researchers and public health practitioners to investigate the underlying motives for and barriers to physical activity behaviour (PAB) [8–10]. Understanding these aspects is crucial for reversing such trends and mitigating their associated health risks. Research in this area has identified numerous determinants of PAB [11], which are commonly categorized into individual, biological [12], environmental [13], psychological [14], behavioural [15], and socio-cultural [16–18] factors.

However, a persistent challenge in PAB research lies in the inconsistent definitions of its determinants [19]. For instance, “parental modelling” has been frequently mentioned in the literature as a determinant that influences PAB in youth; yet there are at least four distinct definitions of this term, including: (1) “behaviours that parents engage in to form and maintain interpersonal relationships with other adults, namely their spouses, relatives, and friends” [20]; (2) “a process of observational learning in which the parent's behaviour serves as a stimulus for similar behaviour in their child” [21]; (3) “the idea that parents' physical activity behaviours may directly influence their children's physical activity” [22]; and (4) “parents acting as positive physical activity role models by demonstrating an interest in physical activity and being physically active themselves” [23]. Such inconsistencies in terminology pose a significant obstacle in advancing research and promoting positive PAB; they also hinder comparing between findings across studies, conducting synthesis of evidence, and developing reliable measurement tools. As such, achieving unification and international consensus for these definitions is essential.

To address these obstacles, the aim of this study was to establish a consensus regarding the definitions of key determinants that are highly associated with PAB. By harmonizing the terminology in this field, this effort seeks to enhance the comparability and relevance of future PAB research, fostering clearer communications and collaborations among researchers, practitioners, policymakers, and other stakeholders worldwide.

## Methods

### Study design

**Fig. 1:**
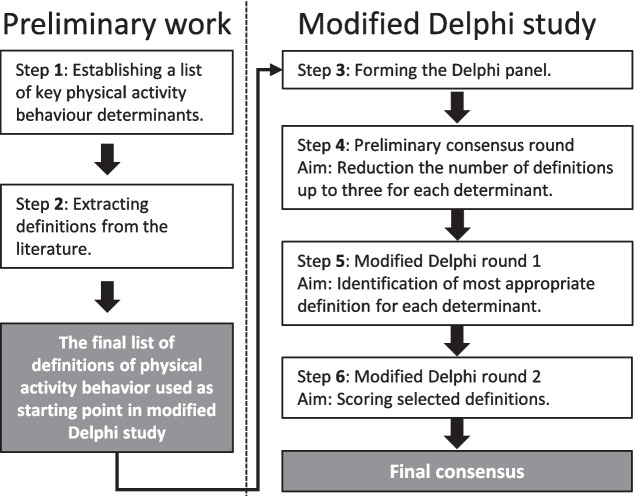
Overview of the preliminary work (Phase 1) and the modified Delphi study (Phase 2)

This consensus work was conducted under Work Group 3 of the European Cooperation in Science and Technology (COST) Action CA19101, “DEterminants of Physical ActivitieS in Settings” (DE-PASS, https://depass.eu/), involving academic and practitioner experts in the fields of sport, health, psychological, and social sciences. The methods were structured into two main phases: preliminary work and the modified Delphi study, as outlined by Niederberger & Spranger [24]. Figure 1 illustrates the sequential stages of both the preliminary work (Steps 1–2) and the modified Delphi study (Steps 3–6). A protocol was developed and registered a priori on the Open Science framework [25].

### Phase 1: the preliminary work

The two steps that comprised this phase were conducted by the steering committee, as detailed below.

#### Step 1. Establishing a list of key PAB determinants

From October to December 2021, the six steering committee experts examined the conclusions of recent academic research articles from peer-reviewed journals, with an emphasis on umbrella and systematic reviews [10, 12–18, 26–28]. One main source was the DEterminants of DIet and Physical ACtivity (DEDIPAC), a European joint-programme initiative [19]. Determinants were also selected based on the preliminary results of a recently published systematic review and meta-analysis [29], conducted by members of the DE-PASS project. Following this process, the six steering committee experts examined and discussed all extracted determinants. The 41 final determinants were then systematically categorized into one of the following five domains:

1. Individual and biological factors: (1) age; (2) body fat; (3) education attainment level; (4) ethnicity; (5) genetic profile and regulation; (6) health status; (7) heart rate; (8) household income; (9) life events; (10) physical fitness; (11) setting; (12) sex; (13) socio-economic status; and (14) stress.
2. Psychological factors: (1) enjoyment; (2) mental fatigue; (3) motivation / goal setting; (4) parental (role) modelling; (5) perceived behavioural control; (6) perceived competency; (7) self-efficacy; and (8) self-regulation.
3. Behavioural factors: (1) active transport; (2) independent active mobility; (3) PAB history and patterns; (4) participation in organised sports; (5) phone usage; (6) sedentary behaviour; and (7) sleep.
4. Environmental factors: (1) access to sport/recreational facilities; (2) availability of physical activity programmes and equipment within schools and community; (3) backyard access/size; (4) green space access; (5) neighbourhood characteristics; (6) physical activity provision and ethos in the setting; (7) provision proximity (parks/playground); and (8) time outdoors.
5. Socio-cultural factors: (1) companionship; (2) cultural perspectives on PAB; (3) group, family and peer support; and (4) social contact.

#### Step 2. Extracting definitions from the literature

To identify the existing definitions of the 41 determinants, a comprehensive search was conducted in PubMed. The search strategy employed the proximity search feature, as a means for locating articles where the terms of the determinants (e.g., age, sex, socio-economic status) combined with the words “defined,” “define,” “defines,” “defining,” or “describe” were within three words of each other in the title or abstract; to do so, the following search query was applied: [Title/Abstract: ~ 3]. This was conducted discretely for each determinant. Additionally, phrase searching using quotation marks was performed via Ovid MEDLINE databases and the Google Scholar search engine. The determinants were searched together with phrases such as “refers to”, “is defined,” “was defined,” “definition of,” “define,” “defines,” “defining,” “is described,” or “was described.” The search was limited to articles written in English, with the first 500 results being screened, ordered by relevance.

Two of the steering committee experts (JD and JB-S) screened the retrieved articles independently, while also seeking potential definitions of the determinants – by manually searching the Medical Subject Headings, dictionaries, policy documents, websites, and reports of organisations related to sports, health, psychology, and social sciences. All definitions were organized in a structured table format, together with their corresponding references. In cases where no definition was found, the determinant from the list of 41 items was still included in the modified Delphi study, detailed below.

### Phase 2: the modified Delphi study

This phase included four steps, as detailed in the following sections.

#### Step 3. Forming the Delphi panel

After creating a comprehensive list of 41 determinants and their definitions, the committee approached 92 members of the COST Action CA19101, inviting them to take part in the Delphi rounds (steps 5 and 6) of this study. These members were from a wide range of disciplines, work settings (e.g., universities, government offices, and hospitals), and countries (more than 30). Active members from diverse scientific backgrounds and at various career stages were invited to form a steering committee. The committee was composed of six international experts (JD, JB-S, JC, MKG, JCR, and YN) — three men and three women — from different countries: Portugal (*n* = 2), Spain (*n* = 1), Norway (*n* = 1), Czechia (*n* = 1), and Israel (*n* = 1). The committee met on a bi-monthly schedule.

#### Step 4. Preliminary consensus round

To streamline the definitions identified from the literature, a preliminary consensus round was conducted in October 2022, with the participation of five of the steering committee members. The sixth member (JB-S), who chaired the procedure, did not participate – to maintain independence and objectivity. For each of the 41 determinants, the five members individually rated the definitions on a scale of 1 (worst definition) to 3 (best definition), according to the following four criteria: (1) universal applicability; (2) broad relevance; (3) clarity; (4) and comprehensibility. The scores for each definition were then summed and ranked in descending order; the top three definitions for each determinant were then selected to be used in Step 5. In the event of a tie between definitions, these were discussed among the committee experts until consensus was reached, resulting in three highest-scoring definitions. Where fewer than three definitions were identified for a particular determinant, this was explicitly stated. In this preliminary round, the a-priori threshold for consensus between the five experts was set at 100%.

#### Step 5. Modified Delphi round 1

Conducted during February–April 2023, the aim of this round was to select the most appropriate definition for each of the 41 PAB determinants, based on the preliminary work of the steering committee. An online survey via Google forms (Google, USA) was distributed to the panel members. Complete anonymity and confidentiality were assured to all participants. After providing their informed consent via this online platform, socio-demographic data were gathered, including age, sex, academic qualifications, and country of residence. Next, the 41 determinants were presented, each with the three optimal definitions. These were organized into the five categories detailed above (individual and biological; psychological; behavioural; environmental; and socio-cultural). The members were then asked to select the one definition that they perceived as most suitable for each determinant. If no definition was deemed suitable, or if the definitions were lacking, the members could suggest an alternative definition, supported by a reference. Comments regarding the definitions were also encouraged. Alternatively, the members could select the following option: “I do not want to answer this question,” if, for example, the concept was outside their area of expertise.

After completing the modified Delphi round 1, each of the six steering committee experts analysed the collected data independently. If a definition was widely accepted (by ≥ 75% among the panel members), it was automatically included in the next stage of the study. If a definition was deemed unsuitable by the panel members (< 75% acceptance), the steering committee refined its wording, based on the highest-ranked definition from the survey and on the members’ feedback, if available. When the gap between the two highest rating definitions was relatively small (< 10%), the committee considered combining elements from multiple definitions, to improve clarity and relevance. During this individual analysis process, the committee experts also examined the alternative definitions that were provided by the panel members, as well as any other comments that they may have offered. In this round and in the following one, the a-priori threshold for consensus between the panel members was set at ≥ 75% [30].

#### Step 6. Modified Delphi round 2

In August–September 2023, an online survey via Google forms (Google, USA) was distributed to the same experts as in round 1. Similar to Step 5, the members provided their informed consent to take part in this survey, as well as socio-demographic data. Complete anonymity and confidentiality were again assured to all participants. In this round 2, the 41 determinants were presented to the panel members – each with the most suitable definition, resulting from round 1. For each determinant, the members were asked to rate the suitability of the given definition, on a Likert-like scale from 1 (strongly disagree) to 5 (strongly agree). The a-priori threshold for consensus was set at 75%, whereby at least 75% of the members had to agree strongly (5) or somewhat agree (4) with a particular definition. The results were then categorised into two groups: (1) determinants that met the 75% threshold, and which were presented during the final steering committee meeting for concluding revisions; and (2) determinants that did not meet this threshold.

### Revision of the definitions and final consensus

The concluding steering committee meeting was moderated by the leader of DE-PASS Deliverable D3.3. Input from round 2 was consolidated, and revisions of the definitions were made as needed. The aim was to compile a conclusive list of agreed-upon definitions for the 41 determinants. During this meeting, the six experts individually evaluated each of the proposed definitions from Step 6, making minor refinements as needed – to standardize the English usage and ensure a consistent academic tone. At this stage, a professional English language expert was engaged, to ensure clarity, coherence, and high-quality English. Definitions that did not meet the agreement threshold in Step 6 were excluded from further analysis.

## Results

### Preliminary consensus round

In this round, at least three definitions were available for 34 of the 41 determinants. For the remaining seven determinants, only two or fewer definitions were available: (1) genetic profile and regulation; (2) backyard access/size; (3) physical activity provision and ethos in setting; (4) PAB history and patterns; (5) access to sport/recreational facilities; (6) availability of physical activity programmes and equipment within schools and communities; and (7) cultural perspectives on PAB. Following this round, a total of 117 definitions were included in the modified Delphi Round 1, including 107 definitions from the expert’s review of the literature and ten that were proposed by the committee experts and supported by references. The most frequent source of definitions in this preliminary consensus round were the MeSH database (Medical Subject Headings) with 16 definitions, the APA Dictionary of Psychology with 15 definitions, and a Dictionary of Public Health with 13 definitions (see Additional file 1).

#### Characteristics of the experts in the modified Delphi rounds

Out of the original 92 members who were invited to participate in the modified Delphi rounds, 85 members participated in the first modified Delphi round, and 69 experts participated in the second one. Table 1 presents the socio-demographic characteristics of these members. Sex representation was approximately equal across the sample. The most highly represented age category was 25–39 years in rounds 1 and 2 (42.4% and 36.1%, respectively). Additionally, most members were affiliated with a university and held a doctoral degree. The members were from a total of 32 countries, highlighting diverse international representation in this study.

**Table 1:** Characteristics of the Delphi panel members in rounds 1 and 2

	**Round 1 (*n* = 85)**		**Round 2 (*n* = 69)**	
**Sex**	*n*	%	*n*	%
Male	44	52	36	52
Female	41	48	33	48
**Age group (years)**				
25–39	36	42	25	36
40–49	22	26	20	29
50 +	27	32	24	35
**Work setting**				
University	76	90	58	86
Government office	4	5	5	7
Other	5	5	6	7
**Level of Education**				
Doctorate	69	81	54	78
Ph.D. student	11	13	10	16
Master’s student	3	3	3	4
Other	2	3	2	3

#### Modified Delphi round 1: identifying the optimal definitions

In this round, levels of agreement between the 85 panel members varied greatly. For 24 determinants, 50% agreed on the same definition. For 13 determinants, 50%–74% agreed on the same definition. The consensus threshold of ≥ 75% was only achieved for definitions of four determinants: (1) neighbourhood characteristics, 75.3%; (2) cultural perspective on PABs, 76.5%; (3) access to sport/recreational facilities, 78.8%; and (4) availability of physical activity programmes and equipment within schools and community, 88.2%. Finally, for 33 determinants, at least one member provided a comment or proposed an additional/alternative definition from the literature.

The committee experts decided that the definitions for the following eight determinants would remain unchanged in round 2: (1) socio-economic status; (2) education attainment level; (3) setting; (4) physical fitness; (5) self-efficacy; (6) sedentary behaviour; (7) sleep; and (8) social contact. Definitions for 32 determinants were modified, mostly involving minor changes – such as the use of British English, avoiding exclusive focus on specific age groups or geographic areas, and adjusting the length of these definitions. Based on expert feedback, a new definition for one determinant, companionship, was established. The final version of all definitions following round 1 is available in Additional file 2.

#### Modified Delphi round 2: identifying the definitions with the highest score

In this round, the best definition for each determinant was identified. The required ≥ 75% consensus threshold was seen for 39 of the 41 determinants (mean score = 88.4%). The highest agreement (98.6%) was seen for educational attainment level. This threshold was not achieved for two determinants: (1) genetic profile and regulation (69.6%); and (2) backyard access/size (73.9%).

#### Final consensus

The steering committee held an online meeting to discuss and finalize the definitions of the 39 determinants for which consensus had been reached (see Additional file 3). Table 2 exemplifies how the final definition of one determinant (age) evolved throughout the two rounds of the modified Delphi study.

**Table 2:** Evolution of a definition of a PAB determinant—age

Phase	Definition
Round 1	The amount of time elapsed since an individual’s birth, typically expressed in terms of months and years. Also called chronological age.
Round 2	The span of time since a person's birth, generally marked in years or months. Also known as chronological age.
Final definition	The span of time since an individual’s birth, generally marked in years or months. Also referred to as chronological age.

Most of the changes, referred to as linguistic refinements, involved adding missing indefinite articles, changing prepositions, modifying singular and plural forms, altering the word order, or unifying terms such as “person” and “individual” across definitions. As mentioned, a professional English language editor assisted the steering committee, to ensure the uniform use of British English and an academic tone across all definitions. The original wording was only maintained for four definitions: (1) life events, derived from the MeSH term “life change events” [31]; (2) sedentary behaviour [32]; (3) self-efficacy [33]; and (4) sleep [34]. For 11 determinants, new definitions were established following the modified Delphi study: (1) setting; (2) PAB history and patterns; (3) phone usage; (4) participation in organised sports; (5) green space access; (6) physical activity provision and ethos in setting; (7) provision proximity (parks/playground); (8) access to sports/recreational facilities; (9) time outdoors, availability of physical activity programmes and equipment within schools and the community; (10) cultural perspective on PABs; and (11) group, family, and peer support. The consensus on the final versions of definitions is provided in Table 3.

**Table 3:** The final version of the definitions for the PAB determinants

	**Agreement (%)**^a^	**Final definition**^**1**^
*Individual and biological*		
Age	**95.7**	The span of time since an individual’s birth, generally marked in years or months. Also referred to as chronological age. (Based on APA Dictionary of Psychology [35], modified)
Body fat	**88.4**	Specialised tissue known as adipose, with distinctive metabolic and endocrine functions, and often addressed within the context of body composition. (Based on Sebastiano [36], modified)
Education attainment level	**98.6**	The highest level of schooling or education completed by an individual or group. (Based on APA Dictionary of Psychology [37], modified)
Ethnicity	**85.5**	A shared identity that distinguishes between different subgroups, based on a common geographical or national origin, and rooted in cultural, historical, religious, and traditional facets. (Based on Barkan [38], modified)
Genetic profile and regulation	69.6	The agreement did not reach the required threshold.
Health status	**78.3**	The extent to which an individual or group can perform or engage in anticipated roles and functions, at a physical, mental, emotional, and social level. (Based on Porta & Last [39], modified)
Heart rate	**92.8**	The frequency at which an individual’s heart contracts within a specific time period, typically per minute. (Based on Zipes [40], modified)
Household income	**85.5**	The aggregate income of all members of a household over a specific time period, adjusted for the number of members and the overall household size. (Based on Lee et al. [41], modified)
Life events	**87.0**	Social, psychological, and environmental occurrences that require adaptation or trigger a change in an individual's pattern of living. Based on National Center for Biotechnology Information [31], modified)
Physical fitness	**85.5**	An individual's capacity to perform physical activities, including components such as cardiorespiratory fitness, musculoskeletal fitness (i.e., muscular endurance and strength), flexibility, and body composition. (Based on Caspersen et al. [42], modified)
Setting	**92.8**	A specific environment or context in which particular behaviours are observed. (Established during the study)
Sex	**89.9**	Biological and physiological distinctions, typically involving variations in reproductive systems, chromosomal patterns, and hormone profiles, most often classified as male or female. (Based on Gender Equality Commission [43], modified)
Socioeconomic status	**94.2**	The position of an individual or group within society, based on a combination of education, income, occupation, and other social factors. (Based on APA Dictionary of Psychology [44], modified)
Stress	**87.0**	A physiological or psychological reaction to intense physical, mental, or emotional demands or challenges, stemming from internal or external sources. (Based on APA Dictionary of Psychology [45], modified)
*Psychological*		
Enjoyment	**92.8**	A subjective experience that is characterised by a sense of pleasure and enthusiasm that is derived from that experience. (Based on Kruk et al. [46], modified)
Mental fatigue	**91.3**	A state of exhaustion and decreased cognitive performance, often associated with prolonged mental activities or stress. (Based on APA Dictionary of Psychology [47], modified)
Motivation / goal Setting	**92.8**	The driving force that instils purpose or direction in an individual’s behaviour, at both a conscious and unconscious level. (Based on APA Dictionary of Psychology [48], modified)
Parental (role) modelling	**92.8**	A learning process through observation, where the behaviour exhibited by the parent serves as a stimulus for similar interests or behaviours in their child. (Based on Tibbs et al. [21], modified)
Perceived behavioural control	**94.2**	The degree to which an individual believes they have active control of their behaviours. (Based on APA Dictionary of Psychology [49], modified)
Perceived competency	**92.8**	An individual's conviction about their ability to perform a given task in an effective and efficient manner. (Based on APA Dictionary of Psychology [50], modified)
Self-efficacy	**92.8**	An individual’s subjective perception of their capability to perform in a given setting or to attain desired results [51]
Self-regulation	**84.1**	An individual's ability to manage and monitor their emotions, behaviours, and desires subject to external demands, as a means for functioning within society. (Based on the National Center for Biotechnology Information [52], modified)
*Behavioural domain*		
Active transport	**91.3**	Performing physical activity as a mode of transport, such as walking, cycling, and other non-motorised means. (Based on French et al. [53] modified)
Independent active mobility	**89.9**	The individual’s ability to physically move around within their environment without assistance. (Based on Wales et al. [54], modified)
PABs history and patterns	**82.6**	An individual's historical engagement in physical activity and their consistent habits, as identified over a specific time period. (Established during the study)
Participation in organised sports	**89.9**	The frequency, duration, and intensity of physical activity performance that entails predefined rules, formal training, and competitions, and that is held by a formal sports organisation. (Established during the study)
Phone usage	**84.1**	The frequency and duration of mobile phone use. (Established during the study)
Sedentary behavior	**92.8**	Any waking behavior characterized by an energy expenditure ≤ 1.5 metabolic equivalents (METs), while in a sitting, reclining or lying posture [32]
Sleep	**92.8**	A circadian state characterized by partial or total suspension of consciousness, voluntary muscle inhibition, and relative insensitivity to stimulation [55]
*Environmental domain*		
Access to sports / recreation facilities	**87.0**	The accessibility and closeness of the nearest sports or recreational facility to the individual’s home or work. (Established during the study)
Availability of physical activity programs and equipment within schools and the community	**84.1**	The existence and accessibility of physical activity initiatives and equipment within educational institutions and local community settings. (Established during the study)
Backyard access/size	73.9	The agreement did not reach the required threshold.
Green space access	**89.9**	The accessibility and closeness of a natural environment in relation to the individual’s place of residence or work (e.g., a school, office, or care facility). (Established during the study)
Neighbourhood characteristics	**89.9**	The demographic, social, architectural, or economic attributes of a geographic area in which individuals reside. (Based on the National Center for Biotechnology Information [56], modified)
Physical activity provision and ethos in setting	**82.6**	The opportunities available to the individual for engaging in physical activity within a given environment. (Established during the study)
Provision proximity (parks / playground)	**92.8**	The geographical distance between a given location and recreational green spaces (e.g., parks or playgrounds). (Established during the study)
Time outdoors	**82.6**	The duration spent engaging in sports and other leisure activities outside of enclosed structures. (Established during the study)
*Socio-cultural domain*		
Companionship	**85.5**	The physical presence of a companion or friend, who tends to provide emotional and social support. (Based on Doble & Santha [57], modified)
Cultural perspective on PABs	**87.0**	The impact of historical and societal factors on the individual’s attitudes, beliefs, motivations, and practices regarding physical activity. (Established during the study)
Group, family, peer support	**91.3**	An individual's perceptions of receiving care, encouragement, and value from their family, friends, colleagues, or others. (Established during the study)
Social contact	**89.9**	Interactions with others, which involve face-to-face or media-related activities. (Based on Taguchi et al. [58], modified)

^a^Percentage of experts reporting “Strongly agree” or “Somewhat Agree” in the modified Delphi round 2

^1^Final version of definition resulted from Final consensus of the Steering Committee

Bold % corresponds to the achievement of the ≥ 75% threshold for agreement; PABs – physical activity behaviors

## Discussion

The aim of this study was to articulate uniform terminology regarding key PAB determinants. This was conducted by examining and synthesising diverse definitions from the literature and incorporating the perspectives of cross-disciplinary experts from more than 30 countries – building on the approach seen in previous studies. When investigating PAB, the absence of systematic and consistent terminology undermines the methodological quality of related studies, complicates the clear interpretation and generalisability of findings, and hinders the translation of knowledge into effective policies and active living intervention strategies [59]. The systematic modified Delphi process applied in this study, and enhanced by expert input, led to high levels of agreement regarding PAB definitions, effectively bridging cultural and interdisciplinary differences. The finalised definitions of the 39 determinants offer a robust foundation for adopting a common language for conducting research on PAB.

Yet consensus was not reached on the definitions of two determinants: (1) genetic profile and regulation; and (2) backyard access/size. Over the past decade, researchers have hypothesised that genetic variations may influence the propensity for physically active lifestyles. Yet the role of these genotype variations in exercise adaptations remains unclear, as the identifying of specific genetic factors which are linked to diverse responses towards physical activity is limited [10, 60]. The lack of consensus regarding the term “genetic profile and regulation” likely stems from the lack of a clear definition of this term prior to conducting related studies. The steering committee experts propose the alternative “polygenic score” [61] term, which, although not widely used or clearly defined in the PAB literature, has potential to serve as an alternative PAB determinant and should be examined in future studies. For the second term that lacked consensus regarding a suitable definition – “backyard access/size” – about 20% of the panel members in round 1 wrote that they do not wish to answer this question. This indicates a degree of uncertainty or lack of familiarity with this term, despite its use in the scientific literature [62, 63]. The term “backyard” may have been too specific, overlooking the large variability in housing configurations. Terms such as “yard” or “private green space” [64] are likely to be more inclusive.

Generally, for all definitions of determinants that contained the word “access” or “accessibility”, the panel members noted inconsistencies in how the term “access” or “accessibility” was applied. Specifically, they suggested that "access" or “accessibility” for private purposes (e.g. backyard) should be defined in binary terms, simply indicating whether an individual “has access” or “does not have access.” In contrast, when these terms refer to public areas (e.g. green space or sports/recreational facilities), the definition should incorporate a specific measure of distance, time, desirability, or safety (e.g., perimeter, walking time, or perceived quality) to enhance clarity and precision.

### Strengths and limitations

The primary strength of this study lies in its geographically diverse and interdisciplinary group of Delphi participants, representing 32 countries and numerous fields of research and practice, with participation in both rounds far exceeding the recommended minimum for achieving consensus in Delphi studies [65]. Defining the agreement thresholds and methods a priori is another notable strength, as this approach is not prevalent in studies – as reported in a systematic review [30]. However, some limitations of this study must be acknowledged. Since the study employed a modified Delphi method, it mainly relied on a preliminary list of PAB determinants and their definitions, as derived from the literature; yet this might have introduced some level of bias. However, if such bias did occur, it was likely overcome by allowing the panel members to add missing definitions for subsequent rounds. In addition, certain determinants do not easily align with a single domain category, for example “physical activity provision and ethos in setting”. While the provision aspect reflects the opportunities available within a given setting (i.e., the environmental domain), the ethos component, which captures the values and cultural context shaping these opportunities [66–69], may conceptually align with the socio-cultural domain. The classification into these domains was carried out primarily to enhance readability and survey feasibility, rather than to suggest rigid conceptual boundaries, and we do not anticipate that this classification approach will affect the establishment of consensus on the definitions. Finally, the list of determinants was mainly based on studies published prior to 2020. Consequently, newer determinants, such as those stemming from government restrictions in light of the COVID-19 pandemic [70], addiction to social networks [71], the global increase in the number and intensity of war conflicts [72] in recent years, and the growing impacts of climate change [73] were not considered in this study.

## Implications and future research

When considering the multifaceted PAB phenomenon, adopting the standardised definitions proposed in this study across diverse contexts could greatly contribute to research comparability and generalisability, while enhancing communications within the research community. Future research should prioritise the continuous updating and refinement of these definitions, while incorporating emerging determinants related to global events. Furthermore, researchers should explore the applicability of these definitions across different cultural and socio-economic contexts and settings, to ensure the generation of harmonised, reliable, and comparable data. Such efforts would provide a robust basis for future policies and interventions, while ensuring that the definitions remain broadly relevant and useful for capturing the dynamics of the PAB phenomenon.

## Conclusions

Through the modified Delphi approach conducted in this study, we achieved consensus on the definitions of 39 out of 41 determinants. The applied iterative process, which incorporated feedback from a large and diverse group of professionals as well as a linguistic specialist, resulted in the development of robust and standardised definitions. These definitions could significantly contribute to the field of PAB – in both theory and practice. The harmonised terminology could significantly enhance the comparability of research findings, advance practice, and foster more effective communications and understandings within the PAB community of researchers and practitioners.

## Supplementary Information

Additional file 1. Preliminary round definitions.

Additional file 2. Round 1 and round 2 definitions.

Additional file 3. Final definitions.

## Data Availability

Survey materials and full lists of definitions are available in supplementary materials.

## Abbreviations

COST: Cooperation in Science and Technology
DEDIPAC: Determinants of Diet and Physical Activity
DE-PASS: Determinants of Physical Activities in Settings
PAB: Physical activity behaviour
