# Supplementary material for: Definitions of determinants of physical activity behaviour: process and outcome of consensus from the DE-PASS expert group
Source: Int J Behav Nutr Phys Act. 2025 Mar 18;22:34. doi: 10.1186/s12966-025-01728-5 (PMC11921651; doi:10.1186/s12966-025-01728-5)
Supplement: Supplementary file 1 — Additional file 1. Preliminary round definitions. [file 12966_2025_1728_MOESM1_ESM.docx]

| Supplementary File 1: Preliminary Round Definitions | | | |
| --- | --- | --- | --- |
| Determinant | **Definition** | | |
|  | **1** | **2** | **3** |
| Age | The duration of time a person has lived. Age is conventionally defined from the time of birth, which counts as zero, and is measured in completed years of life. For some purposes, age is measured from conception, and sometimes it may be convenient to use units shorter than a year [1]. | The amount of time elapsed since an individual’s birth, typically expressed in terms of months and years. Also called calendar age [2]. | Age is defined in full years at the time of survey response [3]. |
| Sex | Sex refers to a person's biological status and is typically categorized as male, female or intersex. There are a number of indicators of biological sex, including sex chromosomes, gonads, internal reproductive organs and external genitalia [4]. | Sex refers to the different biological and physiological characteristics of males and females, such as reproductive organs, chromosomes, hormones, etc [5]. | Sex was defined as male and female [6]. |
| Socioeconomic status (SES) | A descriptive term for the position of persons in society, based on a combination of occupational, economic, and educational criteria, usually expressed in ordered categories, that is, on an ordinal scale. Many classification systems have been proposed, from a simple division according to occupation, which usually relates closely to income and educational level, to more complex systems based on specific details of educational level, income, occupation, and sometimes other criteria, such as whether the usual place of dwelling is owned or rented and the ratable value of the dwelling. Other factors, including ethnicity, literacy, and cultural characteristics, influence socioeconomic status, which is an important determinant of health [7]. | The position of an individual or group on the socioeconomic scale, which is determined by a combination of social and economic factors such as income, amount and kind of education, type and prestige of occupation, place of residence, and—in some societies or parts of society—ethnic origin or religious background [8] | Socioeconomic status is the social standing or class of an individual or group. It is often measured as a combination of education, income and occupation. Examinations of socioeconomic status often reveal inequities in access to resources, plus issues related to privilege, power and control [9]. |
| Ethnicity | Ethnicity refers to the shared social, cultural, and historical experiences, stemming from common national or regional backgrounds, that make subgroups of a population different from one another [10]. | A term for the ethnic group to which people belong. Usually, it refers to group identity based on culture, religion, traditions, and customs. In some contexts, it is a “politically correct” term equivalent to the word “race,” which may have pejorative associations [11]. | A group of people with a common cultural heritage that sets them apart from others in a variety of social relationships [12]. |
| Education attainment level | Educational attainment refers to the highest level of education completed by a person, shown as a percentage of all persons in that age group [13]. | The highest level of schooling completed. Research has linked higher educational attainment levels to greater income level, better health, and other positive outcomes [14]. | The status of learning that has been achieved by a student or group of students [15]. |
| Setting | A home/household setting encompasses a person living alone or a group of people living in the same dwelling, be it a family, several families or unrelated individuals of all ages [16]. | A set is a small close-knit group of people, e.g., a teenage gang, who behave similarly, have similar values, and mutually reinforce one another to maintain these values and behavior. A setting is the social milieu in the context of which particular behaviors are observed. The combination of set and setting is a determinant of many aspects of social conduct, especially in adolescence, including health-related customs and behavior [17]. | School/Education settings include schools and school-boards with childcare, elementary, secondary and postsecondary divisions. Education settings reach a large proportion of the Canadian population—infants, toddlers, preschoolers, young children, youth and young adults—and indirectly reach others—siblings, parents, grandparents, caregivers, teachers, administrators, etc. Work/Workplace settings = Workplace settings include all the organizations that employ Canadians—which amounts to a substantial proportion of the population. Workplaces can strive to ensure that their mandates, strategic plans, operating practices and external messages encourage employees and others to optimize movement behaviours. Community service settings = Community service settings include staff, coaches, instructors and administrators at physical activity, child/youth services and community organizations that offer programs and services to Canadians of all ages (e.g. after-school programs, arts and recreation programs, national and provincial sport organizations, local community centres) [18]. |
| Household income | Gross total annual household income was defined as the sum of all household members' self-reported incomes in euro, divided by the square root of the household size and dichotomized according to the country-specific poverty threshold [19]. | Household income was defined as the average monthly gross income divided by an equivalence factor (equal to the number of household members×0.5) to adjust for differences in household size and composition [20]. | The current household income was defined as the total income of the family per month  [21]. |
| Health Status | The degree to which a person (or specified group) can fulfil usually expected roles and functions physically, mentally, emotionally, and socially. Any departure from the usually expected status is an indication that disease is present. Health status can be assessed clinically by asking questions and conducting a physical examination and/or by health status indicators or health measurement scales [22]. | Health status was defined as an individual’s perceived impact of disease on daily function and well-being in physical, mental, and social domains of health [23]. | The level of health of the individual, group, or population as subjectively assessed by the individual or by more objective measures [24]. |
| Stress | An environmental, social, behavioral factor or combination of factors capable of inducing responses that are helpful in fight-or-flight survival situations, desirable in moderation to provide incentives but under some conditions may be harmful to physical, mental, and/or emotional health by destabilizing the neural, psychological, and/or endocrine equilibrium of the body. When the human body is exposed to stress, psychological, neurological, metabolic, and hormonal reactions are induced. Prolonged or repeated exposure to stress causes lasting changes known as the general adaptation syndrome [25]. | The physiological or psychological response to internal or external stressors. Stress involves changes affecting nearly every system of the body, influencing how people feel and behave. For example, it may be manifested by palpitations, sweating, dry mouth, shortness of breath, fidgeting, accelerated speech, augmentation of negative emotions (if already being experienced), and longer duration of stress fatigue. Severe stress is manifested by the general adaptation syndrome. By causing these mind–body changes, stress contributes directly to psychological and physiological disorder and disease and affects mental and physical health, reducing quality of life [26]. | Stress is how your brain and body respond to a challenge or demand. When you are stressed, your body releases chemicals called hormones. The hormones make you alert and ready to act. They can raise your blood pressure, heart rate, and blood sugar levels. This response is sometimes called a "fight or flight" response. Everyone gets stressed from time to time. There are different types of stress. It can be short-term or long-term. It can be caused by something that happens once or something that keeps happening. Not all stress is bad. In fact, it can help you survive in a dangerous situation. For example, one kind of stress is the jolt you may feel when a car pulls out in front of you. This jolt of hormones helps you quickly hit the brakes to avoid an accident. A little short-term stress can sometimes be helpful [27]. |
| Life events | Life course - A culturally defined sequence of stages that people typically pass through as they progress from birth to death. Health across the lifespan reflects a complex interplay of biological, behavioural, psychological, and social protective and risk factors that contribute to health outcomes across the span of a person’s life  [28]. | Life Change Events - Those occurrences, including social, psychological, and environmental, which require an adjustment or effect a change in an individual's pattern of living [29]. | Important occasions throughout the lifespan that are either age-related and thus expected (e.g., marriage, retirement) or unrelated to age and unexpected (e.g., accidents, relocation). Contextual theories of personality often assume that personality is shaped by reactions to stress produced by critical life events [30]. |
| Physical fitness | Physical fitness refers to an individual's capacity to perform physical activity and includes components such as cardiorespiratory fitness, musculoskeletal fitness (i.e., muscular endurance and strength), and flexibility [31]. | Physical fitness is one’s ability to execute daily activities with optimal performance, endurance, and strength with the management of disease, fatigue, and stress and reduced sedentary behavior [32]. | The ability to carry out daily tasks and perform physical activities in a highly functional state, often as a result of physical conditioning [33]. |
| Heart rate | Is the number of beats of the heart per unit of time. Typically it is measured in beats per minute (bpm). Heart rate is based on the time interval between one R wave and the next. The R wave is the onset of ventricular depolarization [34]. | Refers to the number of times a person’s heart beats per minute. The normal heart rate varies from person to person, but the normal range for adults is 60 to 100 beats per minute (BPM) [35]. | The number of times the HEART VENTRICLES contract per unit of time, usually per minute [36]. |
| Body fat | Body fat generally refers to adipose tissue, a complex connective tissue with specific roles in metabolism and endocrine function. While the terms “adipose tissue” and “body fat” can be used synonymously, body fat is most often used in the context of body composition, while adipose tissue is more often used when describing the physiological properties of fat. Fat consists of a variety of different cells including adipocytes (fat storage cells), connective tissue matrix (nonliving material to nourish the cells), nerve tissue, stromal vascular cells, and immune cells [37]. | Adiposity - Overall expansion of body fat, often with depletion of muscle mass, physical strength, and agility. It should be distinguished from *obesity, which is expansion of body mass involving fat tissue, muscles, and viscera. Adiposity is common in affluent societies, where it is usually confused with and called “obesity.” Adiposity is a disorder of lifestyle, whereas obesity often runs in families and may be genetically determined [38]. | Adipose tissue - specialized connective tissue composed of fat cells (adipocytes). it is the site of stored fats, usually in the form of triglycerides. in mammals, there are two types of adipose tissue, the white fat and the brown fat. their relative distributions vary in different species with most adipose tissue being white [39]. |
| Genetic profile and regulation | Genetic Profile - A set of characteristics unique to the DNA of an individual organism or population obtained by analyzing small samples of biological material such as body tissues or fluids, used especially in the context of predicting susceptibility to a particular disease. For example, some populations have identifiable genetic profiles which put them at higher risk for cancer. Distinguish from GENETIC BACKGROUND and GENETIC VARIATION [40]. | Information about specific genes, including variations and gene expression, in an individual or in a certain type of tissue. A genetic profile may be used to help diagnose a disease or learn how the disease may progress or respond to treatment with drugs or radiation [41]. |  |
| Perceived competency | An individual’s belief in his or her ability to learn and execute skills [42]. | Is defined as “the ability of a person to accomplish challenging tasks and having the feeling that he/she can reach his/her goal” [43]. | Is defined as the extent to which a person feels confident about their ability to complete a task, and it reflects the user's perception of whether they can interact with a system effectively to perform a task and gain value outcomes [44]. |
| Self-regulation | Self-Control is an individual's ability to manage and monitor their emotions, behaviors, and desires in the face of external demands in order to function in society [45]. | Is a dynamic and systematic process that involves efforts to modify and modulate thoughts, emotions, and actions in order to attain goals [46]. | Voluntary adherence to accepted standards; e.g., as applied by physicians to the provision of medical care. This method is advocated by conservative governments for industries to police themselves; e.g., to monitor and control their own environmental pollution. This rarely works as well as professional self-regulation because corporations and industries have agendas and goals but seldom have effective codes of conduct or relevant sanctions for violation of codes [47]. |
| Mental fatigue | A condition of low alertness or cognitive impairment, usually associated with prolonged mental activities or stress [48]. | A state of tiredness and diminished functioning. Fatigue is typically a normal, transient response to exertion, stress, boredom, or inadequate sleep but also may be unusually prolonged and indicative of disorder (e.g., chronic fatigue syndrome, anemia, hypothyroidism). 2. reduced response of a receptor cell or sense organ resulting from excessive stimulation [49]. | Exhaustion, associated with overwork, long working hours, strenuous physical work, demanding intellectual work. A condition predisposing to work-related injury. 2. Muscle fatigue, associated with excessive and/or repetitive activity of particular muscle groups [50]. |
| Motivation/goal setting | The impetus that gives purpose or direction to behavior and operates in humans at a conscious or unconscious level (see unconscious motivation). Motives are frequently divided into (a) physiological, primary, or organic motives, such as hunger, thirst, and need for sleep; and (b) personal, social, or secondary motives, such as affiliation, competition, and individual interests and goals. An important distinction must also be drawn between internal motivating forces and external factors, such as rewards or punishments, that can encourage or discourage certain behaviors. 2. in conditioning, the variables, collectively, that alter the effectiveness of reinforcers. Compare establishing operation. 3. a person’s willingness to exert physical or mental effort in pursuit of a goal or outcome. See work motivation. 4. the act or process of encouraging others to exert themselves in pursuit of a group or organizational goal [51]. | Motivation - Those factors which cause an organism to behave or act in either a goal-seeking or satisfying manner. They may be influenced by physiological drives or by external stimuli [52]. | Objective - A precise detailed statement of the aims toward which efforts are directed. The objectives of a program or service may be arranged in general or specific terms or hierarchically [53]. |
| Perceived behavioral control | Behavior Control - Manipulation of the behavior of persons or animals by biomedical, physical, psychological, or social means, including for nontherapeutic reasons [54]. | Perceived behavioral control refers to the factors that may impede the performance of the behavior which can be categorized into two components. The first part is self-efficacy which can be defined as an individual's self confidence in his or her ability to perform a behavior. The second part, on the other hand, termed as facilitating condition, refers to availability of resources needed to engage in a behavior [55]. | The extent to which a person believes a behavior is under his or her active control [56]. |
| Enjoyment | A perception of great pleasure and happiness brought on by success in or simple satisfaction with an activity [57]. | Physical activity enjoyment is defined as “a positive affective state that reflects feelings such as pleasure, liking and fun” when participating in physical activity for children [58]. | Physical activity enjoyment is defined as the subjective experience characterized by the pleasure and enthusiasm involved in its practice [59]. |
| Self-efficacy | Cognitive mechanism based on expectations or beliefs about one's ability to perform actions necessary to produce a given effect. It is also a theoretical component of behavior change in various therapeutic treatments [60]. | An individual’s subjective perception of his or her capability to perform in a given setting or to attain desired results, proposed by Albert Bandura as a primary determinant of emotional and motivational states and behavioral change. Also called perceived self-efficacy [61]. | Is the belief in personal ability to successfully perform challenging life tasks. Self-efficacy plays an important role in a person’s emotions, cognitions, motivational activities, and behaviors across a variety of activities [62]. |
| Parental (role) modeling | Parental modeling is defined as a process of observational learning in which the behavior of the parent acts as a stimulus for similar behavior in his or her child [63]. | Parental modeling refers to parents acting as positive physical activity role models by showing an interest in physical activity as well as being physically active themselves [64]. | Parental modeling refers to idea that parents’ own physical activity behaviours may directly influence their children’s physical activity [65]. |
| PABs history and patterns | PAB history - How physically active a person was in the past (provided by an expert); PAB pattern - A relatively stable schedule in which an individual engages in physical activity (provided by an expert). |  |  |
| Sedentary behaviour | Sedentary behavior was defined as activities involving low energy expenditure, equivalent to 1.0 to 1.5 metabolic equivalent units (METs), such as remaining seated or lying down, reading, watching TV, playing video games, or other forms of entertainment based on a screen [66]. | Sedentary behavior is any waking behavior characterized by an energy expenditure ≤1.5 metabolic equivalents (METs), while in a sitting, reclining or lying posture [67]. | Behaviors during waking hours that have low energy expenditure and are often performed in a sitting or reclining POSTURE [68]. |
| Phone usage | Utilization of phones for communication (measured in minutes-hours) (provided by an expert). | The frequency of duration a phone is used (provided by an expert). | Utilization of wireless phones for communication [69]. |
| Independent active mobility | Independent active travel was defined as habitually walking, riding a bike or skateboard/scooter/rollerblading without adult accompaniment in at least one direction [70]. | Children’s independent mobility (CIM) means the freedom of those under 18 years of age to move in public space without adult accompaniment [71]. | Children’s independent mobility refers to children’s freedom to move around and play in their local environment without adult accompaniment [72]. |
| Sleep | A circadian state characterized by partial or total suspension of consciousness, voluntary muscle inhibition, and relative insensitivity to stimulation. Other characteristics include unique sleep-related electroencephalogram and brain-imaging patterns (see sleep stages). These characteristics help distinguish normal sleep from a loss of consciousness due to brain injury, disease, or drugs [73]. | Sleep is a complex biological process. While you are sleeping, you are unconscious, but your brain and body functions are still active. They are doing a number of important jobs that help you stay healthy and function at your best. So when you don't get enough quality sleep, it does more than just make you feel tired. It can affect your physical and mental health, thinking, and daily functioning [74]. | A readily reversible suspension of sensorimotor interaction with the environment, usually associated with recumbency and immobility [75]. |
| Participation in organised sports | Organized sport is defined as physical activity that is directed by adult or youth leaders and involves rules and formal practice and competition. School and club sports are included in this definition. Physical education classes at schools do not typically fall into the category of organized sport [76]. | The frequency, duration and/or intensity in participating in sport that is organised for a specific purpose such as competition, training (provided by an expert). | Participation in sport activities that are not spontaneously conducted but planned (provided by an expert). |
| Active transport | Active transport (or active commuting) relates to physical activity undertaken as a means of transport and includes walking, cycling and other non-motorised vehicles. It also includes public transport use because this usually involves walking or cycling at the beginning or end of the journey [77]. | Active transport encompasses modes of transport that require the expenditure of human energy, such as walking, cycling, jogging, scootering and skateboarding [78]. | Active transport refers to human-powered forms of travel such as walking and cycling [79]. |
| Backyard access/size | The area in square metres (m^2^) of front, back and/or other yard spaces was reported if known; otherwise parents were instructed to estimate (as accurately as possible) the width and length of their yard space(s) in 1-metre steps [80]. | Backyard access and green space availability within a radius of 300 m [81]. |  |
| Green space access | Access to a major green space larger than 0.5 hectares (5000 m^2^) was measured using straight line distances between home addresses to the boundary of the nearest major green space [82]. | Distance to nearest total green space (km) / Distance to nearest useable green space (km) [83]. | Access to a major green space as ‘living within 300 m of a green space with an area of equal or more than 5000 m^2^ [84]. |
| PA provision and ethos in setting | Low, medium and high physical activity provision was defined as <110 minutes/week, 110–149 minutes per week and ≥150 minutes per week in primary schools; respective values for secondary schools were <150 minutes per week, 150–189 minutes/week and ≥190 minutes/week [85]. | PA provision - the opportunities provided for individuals to be physically active (provided by an expert). |  |
| Neighbourhood characteristics | Characteristics that may describe the demographic, social, built environment, or economic characteristics of a geographic area in which people live and can affect health and well-being of neighborhood residents. Neighborhood environments include the physical environment (unsafe air/water), built environment (sidewalks/greenspace) and the social environment (safety, sense of community) [86]. | A circumscribed locality in which appreciable proportions of the people can be expected to know one another if they have been residents for long enough to get acquainted, such as a “village within a city” or a suburban district [87]. | Neighborhood community design factors that support physical activity include greater residential density, a mix of residential and commercial land uses, and access to schools, parks, and recreation facilities [88]. |
| Provision proximity (parks/playground) | Distance from specific location to parks/playground (provided by an expert). | How close parks/playgrounds are (provided by an expert). |  |
| Access to sport/recreational facilities | Spatial accessibility was defined as the shortest street network distance to the closest sport facility [89]. |  |  |
| Time outdoors | Time spent outdoors was defined as the sum of general activities, leisure activities, and sports performed outdoors [90]. | Time outdoors was defined as hours per day of outdoor physical and leisure activities plus driving and riding in vehicle [91]. | The time spent outdoors was defined as the sum of outdoor leisure and sports [92]. |
| Availability of physical activity programs and equipment within schools and community | The presence of, and how much access people have, to the physical activity programs and equipment in schools and community (provided by an expert). |  |  |
| Cultural perspective on PABs | Historical and cultural perspective to physical activity provides participants the opportunity to value the intent and purpose of movement or physical activity and contemplate how physical activity once again can be enjoyed as an integral part of everyday life [93]. |  |  |
| Group/family/peer support | Peer group support is defined as a system of giving and receiving assistance with respect, collective responsibility, and mutual agreement through support, friendship, empathy, sharing, and mutual assistance [94]. | Group support - A usually informal group of friends, colleagues, etc. Who provide emotional and social support at times of trouble. Epidemiological evidence suggests that the availability of a support group reduces the risk of adverse health outcomes from common serious diseases [95]  Peer support - The form of social bonding and group solidarity whereby peer group members assist one another in adversity [96]. | Peer group support is defined as emotional social support, instrumental support, and sharing in any condition to bring about desired social or personal change. (Repper, J., & Carter, T. (2011). A review of the literature on peer support in mental health services [97]. |
| Companionship | Companionship has been defined as social involvement in shared, activities pursued for the intrinsic goal of satisfaction or enjoyment [98]. | Social companionship is defined as spending time with others in leisure and recreational activities [98]. | Companionship is defined as ‘engagement in activities with others who share common experiences, interests, values or goals’ [99]. |
| Social contact | Interactive social contact refers to face-to-face interactions such as talking with friends, whereas noninteractive social contact involves media-related activities [100]. | A social contact was defined as a face-to-face conversation with more than three words at a distance of less than two meters. The total number of contacts was estimated by adding the reported number of contacts outside the household to the number of individuals living in the household [101]. | Social contact was defined as whenever a child was in company or approached another person or persons or was approached to play, to talk, to listen, etc. (not mere accidental passive standing beside another child or adult) [102]. |

**References:**

1. Porta M, Last JM. Age [Internet]. 2nd ed. A Dictionary of Public Health. Oxford University Press; 2018 [cited 2024 Jul 23]. Available from: https://www.oxfordreference.com/display/10.1093/acref/9780191844386.001.0001/acref-9780191844386-e-88

2. American Psychological Association. Chronological age [Internet]. APA Dictionary of Psychology. [cited 2022 Oct 1]. Available from: https://dictionary.apa.org/chronological-age

3. Juhola J, Arokoski JPA, Ervasti J, Kivimäki M, Vahtera J, Myllyntausta S, et al. Internal consistency and factor structure of Jenkins sleep scale: Cross-sectional cohort study among 80 000 adults. BMJ Open. 2021;11:e043276.

4. American Psychological Association. Guidelines for psychological practice with lesbian, gay, and bisexual clients. American Psychologist. 2012;67:10–42.

5. Council of Europe (Gender Equality Commission). Gender equality glossary [Internet]. 2016. Available from: https://edoc.coe.int/en/gender-equality/6947-gender-equality-glossary.html

6. Mainous AG, Tanner RJ, Anton SD, Jo A, Luetke MC. Physical activity and abnormal blood glucose among healthy weight adults. Am J Prev Med. 2017;53:42–7.

7. Porta M, Last JM. Socioeconomic status (SES). A Dictionary of Public Health [Internet]. 2nd ed. Oxford University Press; 2018 [cited 2024 Jul 23]. Available from: https://www.oxfordreference.com/display/10.1093/acref/9780191844386.001.0001/acref-9780191844386-e-4231?rskey=YZuy9K&result=1

8. American Psychological Association. Report of the APA Task Force on Socioeconomic Status [Internet]. Washington; 2007. Available from: https://www.apa.org/pi/ses

9. American Psychological Association. Socioeconomic status (SES) [Internet]. APA Dictionary of Psychology. [cited 2022 Oct 1]. Available from: https://dictionary.apa.org/socioeconomic-status

10. Barkan SE. Sociology: Understanding and changing the social world. Boston, MA: Flat World Knowledge; 2016.

11. Porta M, Last JM. Ethnicity. A Dictionary of Public Health [Internet]. 2nd ed. Oxford University Press; 2018 [cited 2024 Jul 23]. Available from: https://www.oxfordreference.com/display/10.1093/acref/9780191844386.001.0001/acref-9780191844386-e-1394?rskey=pCC2OX&result=1

12. National Center for Biotechnology Information. Ethnicity. MeSH database.

13. OECD. Education at a Glance 2021 [Internet]. OECD; 2021 [cited 2024 Jul 22]. Available from: https://www.oecd-ilibrary.org/education/education-at-a-glance-2021_b35a14e5-en

14. American Psychological Association. Educational attainment level (2). APA Dictionary of Psychology.

15. American Psychological Association. Educational attainment level (1) [Internet]. APA Dictionary of Psychology. [cited 2022 Oct 2]. Available from: https://dictionary.apa.org/educational-attainment-level

16. Rhodes RE, Guerrero MD, Vanderloo LM, Barbeau K, Barbeau K, Birken CS, et al. Development of a consensus statement on the role of the family in the physical activity, sedentary, and sleep behaviours of children and youth. Int J Behav Nutr Phys Act. 2020;17:1–31.

17. Porta M, Last JM. Setting. A Dictionary of Public Health [Internet]. 2nd ed. Oxford University Press; 2018 [cited 2024 Jul 23]. Available from: https://www.oxfordreference.com/display/10.1093/acref/9780191844386.001.0001/acref-9780191844386-e-4105?rskey=ZaZZcM&result=4

18. Tomasone JR, Janssen I, Saunders TJ, Duggan M, Jones R, Brouwers MC, et al. Timing of 24-hour movement behaviours: ImpIications for practice, poIicy and research. Health Promot Chronic Dis Prev Can. 2022;42:170.

19. Stephan R; Grill E AS. The effect of income and wealth on the onset of health deficit accumulation in older adults in Europe – results from the SHARE study. Das Gesundheitswesen [Internet]. 2017;79:V–48. Available from: http://www.thieme-connect.com/products/ejournals/abstract/10.1055/s-0037-1605645

20. Lee HY, Hahm M Il, Park EC. Differential association of socio-economic status with gender- and age-defined suicidal ideation among adult and elderly individuals in South Korea. Psychiatry Res. 2013;210:323–8.

21. Chao CY, Shih CC, Wang CJ, Wu JS, Lu FH, Chang CJ, et al. Low socioeconomic status may increase the risk of central obesity in incoming university students in Taiwan. Obes Res Clin Pract. 2014;8:e212–9.

22. Porta M, Last JM. Health status. A Dictionary of Public Health [Internet]. 2nd ed. Oxford University Press; 2018 [cited 2022 Oct 5]. Available from: https://www.oxfordreference.com/display/10.1093/acref/9780191844386.001.0001/acref-9780191844386-e-1950?rskey=cveUyY&result=1

23. Stewart AL, Ware JE. Measuring Functioning and Well-being: The Medical Outcomes Study Approach [Internet]. Duke University Press; 1992. Available from: https://books.google.cz/books?id=Wy3dyI_lyb4C

24. National Center for Biotechnology Information. Health Status. MeSH database.

25. Porta M, Last JM. Stress. A Dictionary of Public Health [Internet]. 2nd ed. Oxford University Press; 2018 [cited 2024 Jul 24]. Available from: https://www.oxfordreference.com/display/10.1093/acref/9780191844386.001.0001/acref-9780191844386-e-1950?rskey=cveUyY&result=1

26. American Psychological Association. Stress [Internet]. APA Dictionary of Psychology. [cited 2022 Oct 2]. Available from: https://dictionary.apa.org/stress

27. MedlinePlus. Stress [Internet]. [cited 2024 Jul 22]. Available from: https://medlineplus.gov/stress.html

28. World Health Organization. Health promotion glossary of terms 2021 [Internet]. Geneva; 2021. Available from: https://www.who.int/publications/i/item/9789240038349

29. National Center for Biotechnology Information. Life change events [Internet]. MeSH database. National Library of Medicine; [cited 2022 Oct 5]. Available from: https://www.ncbi.nlm.nih.gov/mesh/68008016

30. American Psychological Association. Life events [Internet]. APA Dictionary of Psychology. [cited 2024 Jul 22]. Available from: https://dictionary.apa.org/life-events

31. Caspersen C, Powell K, Gregory C. Physical activity, exercise, and physical fitness: Definitions and distinctions for health-related research. Public Health Rep. 1985;100:126–31.

32. Campbell N, De Jesus S, Prapavessis H. Physical Fitness. In: Gellman MD, Turner JR, editors. Encyclopedia of Behavioral Medicine [Internet]. New York, NY: Springer New York; 2013. p. 1486–9. Available from: https://doi.org/10.1007/978-1-4419-1005-9_1167

33. National Center for Biotechnology Information. Physical Fitness [Internet]. National Library of Medicine. [cited 2024 Jul 22]. Available from: https://www.ncbi.nlm.nih.gov/mesh/68010809

34. Andreassi JL. Psychophysiology: Human behavior and physiological response. Psychophysiology: Human Behavior and Physiological Response. 2010.

35. Zipes DP. Braunwald’s Heart Disease: A Textbook of Cardiovascular Medicine. 11th ed. Libby P, Bonow RO, Mann DL, Tomaselli GF, editors. Philadelphia: Elsevier Health Sciences; 2018.

36. National Center for Biotechnology Information. Heart Rate. MeSH database.

37. Di Katie Sebastiano M. Body fat. In: Gellman Marc D. and Turner JR, editor. Encyclopedia of Behavioral Medicine [Internet]. New York, NY: Springer New York; 2013. p. 243–5. Available from: https://doi.org/10.1007/978-1-4419-1005-9_1096

38. Porta M, Last JM. Adiposity. A Dictionary of Public Health [Internet]. 2nd ed. Oxford University Press; 2018 [cited 2024 Jul 24]. Available from: https://www.oxfordreference.com/display/10.1093/acref/9780191844386.001.0001/acref-9780191844386-e-58?rskey=XNbyYo&result=3

39. National Center for Biotechnology Information. Adipose Tissue [Internet]. MeSH database. [cited 2024 Jul 22]. Available from: https://www.ncbi.nlm.nih.gov/mesh/68000273

40. National Center for Biotechnology Information. Genetic Profile [Internet]. MeSH database. [cited 2024 Jul 22]. Available from: https://www.ncbi.nlm.nih.gov/mesh/2027854

41. National Cancer Institute. Genetic profile [Internet]. Dictionary of Cancer Terms. [cited 2024 Jul 22]. Available from: https://www.cancer.gov/publications/dictionaries/cancer-terms/def/genetic-profile

42. American Psychological Association. Perceived competence [Internet]. APA Dictionary of Psychology. [cited 2022 Oct 2]. Available from: https://dictionary.apa.org/perceived-competence

43. White RW. Motivation reconsidered: The concept of competence. Psychol Rev. 1959;66:297.

44. Nikou SA, Economides AA. Mobile-based assessment: Investigating the factors that influence behavioral intention to use. Comput Educ. 2017;109:56–73.

45. National Center for Biotechnology Information. Self-control [Internet]. MeSH database. [cited 2022 Oct 4]. Available from: https://www.ncbi.nlm.nih.gov/mesh/2009825

46. Mora PA, Ozakinci G. Self-Regulation Model. In: Gellman MD, Turner JR, editors. Encyclopedia of Behavioral Medicine [Internet]. New York, NY: Springer New York; 2013. p. 1751–6. Available from: https://doi.org/10.1007/978-1-4419-1005-9_983

47. Porta M, Last JM. Self-regulation. A Dictionary of Public Health [Internet]. 2nd ed. Oxford University Press; 2018 [cited 2024 Jul 24]. Available from: https://www.oxfordreference.com/display/10.1093/acref/9780191844386.001.0001/acref-9780191844386-e-4076?rskey=YO0qQe&result=1

48. National Center for Biotechnology Information. Mental Fatigue [Internet]. MeSH database. [cited 2024 Jul 22]. Available from: https://www.ncbi.nlm.nih.gov/mesh/68005222

49. American Psychological Association. Fatigue [Internet]. APA Dictionary of Psychology. [cited 2022 Oct 2]. Available from: https://dictionary.apa.org/fatigue

50. Porta M, Last JM. Mental fatigue. A Dictionary of Public Health [Internet]. 2nd ed. Oxford University Press; 2018 [cited 2024 Jul 24]. Available from: https://www.oxfordreference.com/display/10.1093/acref/9780191844386.001.0001/acref-9780191844386-e-1492?rskey=RPQQvL&result=1

51. American Psychological Association. Motivation [Internet]. APA Dictionary of Psychology. [cited 2022 Oct 2]. Available from: https://dictionary.apa.org/motivation

52. National Center for Biotechnology Information. Motivation [Internet]. MeSH database. [cited 2024 Jul 22]. Available from: https://www.ncbi.nlm.nih.gov/mesh/68009042

53. Porta M, Last JM. Objective. A Dictionary of Public Health [Internet]. 2nd ed. Oxford University Press; 2018 [cited 2024 Jul 29]. Available from: https://www.oxfordreference.com/display/10.1093/acref/9780191844386.001.0001/acref-9780191844386-e-3155?rskey=GaGIpv&result=1

54. National Center for Biotechnology Information. Behavior Control [Internet]. MeSH database. [cited 2024 Jul 22]. Available from: https://www.ncbi.nlm.nih.gov/mesh/68032763

55. Tan M, Teo T. Factors Influencing the Adoption of Internet Banking. J Assoc Inf Syst [Internet]. 2000 [cited 2024 Jul 22];1:1–44. Available from: https://aisel.aisnet.org/jais/vol1/iss1/5

56. American Psychological Association. Perceived behavioral control [Internet]. APA Dictionary of Psychology. [cited 2022 Oct 2]. Available from: https://dictionary.apa.org/perceived-behavioral-control

57. American Psychological Association. Enjoyment [Internet]. APA Dictionary of Psychology. [cited 2024 Jul 22]. Available from: https://dictionary.apa.org/enjoyment

58. Motl RW, Dishman RK, Saunders R, Dowda M, Felton G, Pate RR. Measuring enjoyment of physical activity in adolescent girls. Am J Prev Med. 2001;21:110–7.

59. Kruk M, Zarychta K, Horodyska K, Boberska M, Scholz U, Radtke T, et al. From enjoyment to physical activity or from physical activity to enjoyment? Longitudinal associations in parent–child dyads. Psychol Health. 2018;33:1269–83.

60. National Center for Biotechnology Information. Self Efficacy [Internet]. MeSH database. [cited 2024 Jul 30]. Available from: https://www.ncbi.nlm.nih.gov/mesh/68020377

61. American Psychological Association. Self-efficacy [Internet]. APA Dictionary of Psychology. [cited 2022 Oct 2]. Available from: https://dictionary.apa.org/self-efficacy

62. Butler J. Self-Efficacy. In: Gellman MD, Turner JR, editors. Encyclopedia of Behavioral Medicine [Internet]. New York, NY: Springer New York; 2013. p. 1737–9. Available from: https://doi.org/10.1007/978-1-4419-1005-9_981

63. Tibbs T, Haire-Joshu D, Schechtman KB, Brownson RC, Nanney MS, Houston C, et al. The relationship between parental modeling, eating patterns, and dietary intake among African-American parents. J Am Diet Assoc. 2001;101:535–41.

64. Tremblay MS, Gray C, Babcock S, Barnes J, Bradstreet CC, Carr D, et al. Position statement on active outdoor play. Int J Environ Res Public Health. 2015;12:6475–505.

65. Trost SG, Loprinzi PD. Parental influences on physical activity behavior in children and adolescents: A brief review. Am J Lifestyle Med. 2011;5:171–81.

66. Pate RR, O’Neill JR, Lobelo F. The evolving definition of “sedentary.” Exerc Sport Sci Rev. 2008;36:173–8.

67. Tremblay MS, Aubert S, Barnes JD, Saunders TJ, Carson V, Latimer-Cheung AE, et al. Sedentary Behavior Research Network (SBRN) - Terminology consensus project process and outcome. Int J Behav Nutr Phys Act. 2017;14:1–17.

68. National Center for Biotechnology Information. Sedentary Behavior [Internet]. MeSH database. [cited 2024 Jul 22]. Available from: https://www.ncbi.nlm.nih.gov/mesh/68057185

69. National Center for Biotechnology Information. Cell Phone Use. MeSH database.

70. Veitch J, Carver A, Salmon J, Abbott G, Ball K, Crawford D, et al. What predicts children’s active transport and independent mobility in disadvantaged neighborhoods? Health Place. 2017;44:103–9.

71. Hillman M, Adams J, Whitelegg J. One false move: A study of children’s independent mobility. London; 1990.

72. Wales M, Mårtensson F, Jansson M. “You can be outside a lot”: Independent mobility and agency among children in a suburban community in Sweden. Child Geogr. 2021;19:184–96.

73. American Psychological Association. Sleep [Internet]. APA Dictionary of Psychology. [cited 2022 Oct 2]. Available from: https://dictionary.apa.org/sleep

74. MedlinePlus. Sleep Disorders [Internet]. [cited 2024 Jul 22]. Available from: https://medlineplus.gov/sleepdisorders.html

75. National Center for Biotechnology Information. Sleep. MeSH database.

76. Logan K, Cuff S, Council on Sports Medicine and Fitness, LaBella CR, Brooks MA, Canty G, et al. Organized sports for children, preadolescents, and adolescents. Pediatrics. 2019;143:e20190997.

77. French S, Giles-Corti B, I’Anson K. 2010 UWA commuting survey, volume 1: Executive Summary. Perth; 2010.

78. Cheyne C, Imran M, Scott M, Tien C. Barriers to active transport in Palmerston North [Internet]. Palmerston North; 2015. Available from: www.massey.ac.nz/livinglabs

79. Cole R, Burke M, Leslie E, Donald M, Owen N. Perceptions of representatives of public, private, and community sector institutions of the barriers and enablers for physically active transport. Transp Policy (Oxf). 2010;17:496–504.

80. Armstrong GP, Maitland C, Lester L, Trost SG, Trapp G, Boruff B, et al. Associations between the home yard and preschoolers’ outdoor play and physical activity. Public Health Res Pract. 2019;29:e2911907.

81. Berdejo-Espinola V, Suárez-Castro AF, Amano T, Fielding KS, Oh RRY, Fuller RA. Urban green space use during a time of stress: A case study during the COVID-19 pandemic in Brisbane, Australia. People and Nature. 2021;3:597–609.

82. McEachan RRC, Prady SL, Smith G, Fairley L, Cabieses B, Gidlow C, et al. The association between green space and depressive symptoms in pregnant women: moderating roles of socioeconomic status and physical activity. J Epidemiol Community Health. 2016;70:253–9.

83. Nutsford D, Pearson AL, Kingham S. An ecological study investigating the association between access to urban green space and mental health. Public Health. 2013;127:1005–11.

84. Dadvand P, Wright J, Martinez D, Basagaña X, McEachan RRC, Cirach M, et al. Inequality, green spaces, and pregnant women: Roles of ethnicity and individual and neighbourhood socioeconomic status. Environ Int. 2014;71:101–8.

85. Cleland V, Dwyer T, Blizzard L, Venn A. The provision of compulsory school physical activity: Associations with physical activity, fitness and overweight in childhood and twenty years later. Int J Behav Nutr Phys Act. 2008;5:14.

86. National Center for Biotechnology Information. Neighborhood characteristics. MeSH database.

87. Porta M, Last JM. Neighborhood. A Dictionary of Public Health [Internet]. 2nd ed. Oxford University Press; 2018 [cited 2024 Jul 29]. Available from: https://www.oxfordreference.com/display/10.1093/acref/9780191844386.001.0001/acref-9780191844386-e-3030?rskey=wq2HOZ&result=1

88. Carlson J, Dean K, Sallis J. Measures Registry User Guide: Physical Activity Environment [Internet]. Washington (DC); Available from: http://nccor.org/tools-mruserguides/wp-content/

89. Shrestha S, Kestens Y, Thomas F, El Aarbaoui T, Chaix B. Spatial access to sport facilities from the multiple places visited and sport practice: Assessing and correcting biases related to selective daily mobility. Soc Sci Med. 2019;236:112406.

90. Sherwin JC, Reacher MH, Keogh RH, Khawaja AP, MacKey DA, Foster PJ. The association between time spent outdoors and myopia in children and adolescents: A systematic review and meta-analysis. Ophthalmology. 2012;119:2141–51.

91. Mirhajianmoghadam H, Piña A, Ostrin LA. Objective and Subjective Behavioral Measures in Myopic and Non-Myopic Children During the COVID-19 Pandemic. Transl Vis Sci Technol. 2021;10:4.

92. Zhang X, Yang Y, Zhang S, Zhang H, Yao L, Liu L, et al. TuYou-County Pediatric Eye (TYPE) study, design issues, baseline demographic characteristics, and implications: Report number 1. Medicine. 2021;100:e24670.

93. Ward SA. African-centered dance. J Phys Educ Recreat Dance. 2008;79:4–5.

94. Mead S, Hilton D, Curtis L. Peer support: A theoretical perspective. Psychiatr Rehabil J. 2001;25:134.

95. Porta M, Last JM. Support group. A Dictionary of Public Health [Internet]. 2nd ed. Oxford University Press; 2018 [cited 2024 Jul 29]. Available from: https://www.oxfordreference.com/display/10.1093/acref/9780191844386.001.0001/acref-9780191844386-e-4342?rskey=UlNgwf&result=1

96. Porta M, Last JM. Peer support. A Dictionary of Public Health [Internet]. 2nd ed. Oxford University Press; 2018 [cited 2024 Jul 29]. Available from: https://www.oxfordreference.com/display/10.1093/acref/9780191844386.001.0001/acref-9780191844386-e-3398?rskey=UlNgwf&result=2

97. Solomon P. Peer support/peer provided services underlying processes, benefits, and critical ingredients. Psychiatr Rehabil J. 2004;27:392.

98. Jang J, Kim J. Healthier life with digital companions: Effects of reflection-level and statement-type of messages on behavior change via a perceived companion. Int J Hum Comput Interact. 2020;36:172–89.

99. Doble SE, Santha JC. Occupational well-being: Rethinking occupational therapy outcomes. Can J Occup Ther. 2008;75:184–90.

100. Taguchi N, Xiao F, Li S. Effects of Intercultural Competence and Social Contact on Speech Act Production in a Chinese Study Abroad Context. Mod Lang J. 2016;100:775–96.

101. Latsuzbaia A, Herold M, Bertemes JP, Mossong J. Evolving social contact patterns during the COVID-19 crisis in Luxembourg. PLoS One. 2020;15:e0237128.

102. Bar-Tal D, Raviv A, Goldberg M. Helping behavior among preschool children: An observational study. Child Dev. 1982;53:396–402.
