# Supplementary material for: Definitions of determinants of physical activity behaviour: process and outcome of consensus from the DE-PASS expert group
Source: Int J Behav Nutr Phys Act. 2025 Mar 18;22:34. doi: 10.1186/s12966-025-01728-5 (PMC11921651; doi:10.1186/s12966-025-01728-5)
Supplement: Supplementary file 2 — Additional file 2. Round 1 and round 2 definitions. [file 12966_2025_1728_MOESM2_ESM.docx]

| Supplementary File 2: Definitions resulting from Round 1 and Round 2 | | | |
| --- | --- | --- | --- |
| **No.** | **Determinant** | **Round 1:**  *Revised definition after participants feedback and Steering Committe input* | **Round 2:**  *Revised definition after participants feedback and Steering Committee input* |
| 1 | Age | The amount of time elapsed since an individual’s birth, typically expressed in terms of months and years. Also called chronological age. | The span of time since a person's birth, generally marked in years or months. Also known as chronological age. |
| 2 | Sex | Sex refers to the differences in biological and physiological characteristics, such as reproductive organs, chromosomes or hormones, and is typically categorized as male or female. | Pertains to the biological and physiological distinctions, typically involving variations in reproductive systems, chromosomal patterns, and hormone profiles, most often classified as male or female. |
| 3 | Socioeconomic status (SES) | The position of an individual or group in the society, based on a combination of education, income, occupation and social factors. | The position of an individual or group in the society, based on a combination of education, income, occupation and social factors. |
| 4 | Ethnicity | It refers to group identity based on culture, history, religion, traditions, or customs stemming from common national or regional background that make subgroups of a population different from one another. | A shared identity rooted in cultural, historical, religious, and traditional facets derived from a common geographic or national origin, differentiating certain population subgroups from others. |
| 5 | Education attainment level | The highest level of schooling completed by a person or a group of people. | The highest level of schooling completed by a person or a group of people. |
| 6 | Setting | Specific environment or context within which particular behaviours are observed. | Specific environment or context within which particular behaviours are observed. |
| 7 | Household income | Sum of all household members' incomes divided by an equivalence factor (number of members and household size) over a specific period of time. | The aggregate incomes of all members of a household, adjusted for the number of members and overall household size, over a specific time period. |
| 8 | Health Status | The degree to which a person (or specified group) can fulfill usually expected roles and functions physically, mentally, emotionally, and socially. | The extent to which a person or specified group can engage in and fulfill anticipated roles and functions on physical, mental, emotional, and social levels. |
| 9 | Stress | The physiological or psychological response to internal or external physical, mental or emotional pressure. | A physiological or psychological response to intense physical, mental, or emotional demands or challenges, either from within or external sources. |
| 10 | Life events | Those occurrences, including social, psychological, and environmental, which require an adjustment or effect a change in an individual's pattern of living. | Social, psychological, and environmental occurrences that require adaptation or trigger a change in an individual's pattern of living. |
| 11 | Physical fitness | Individual's capacity to perform physical activity and includes components such as cardiorespiratory fitness, musculoskeletal fitness (i.e., muscular endurance and strength), flexibility and body composition. | Individual's capacity to perform physical activity including components such as cardiorespiratory fitness, musculoskeletal fitness (i.e., muscular endurance and strength), flexibility and body composition. |
| 12 | Heart rate | The number of times a person’s heart beats per minute. | The frequency at which a person's heart contracts within a given time, typically per minute. |
| 13 | Body fat | Adipose tissue with specific roles in metabolism and endocrine function, mostly used in the context of body composition. | Specialized tissue, known as adipose, with distinctive metabolic and endocrine functions, often considered in the context of body composition. |
| 14 | Genetic profile and regulation | A set of characteristics unique to the DNA of an individual or population that can be used to predict susceptibility to or diagnose a disease, learn how the disease may progress, or how it responds to drug or radiation treatment. | A set of characteristics unique to an individual or group's DNA, utilized for predicting disease susceptibility or diagnosis, understanding disease progression, or assessing responses to pharmaceutical or radiation therapies. |
| 15 | Perceived competency | An individual’s belief in his/her ability to perform specific tasks. | A person's conviction about his/her capacity to perform certain tasks effectively. |
| 16 | Self-regulation | An individual's ability to manage and monitor their emotions, behaviors, and desires in the face of external demands in order to function in society. | An individual's ability to manage and monitor their emotions, behaviors, and desires amidst external demands in order to function in society. |
| 17 | Mental fatigue | A state of tiredness and diminished cognitive functioning usually associated with prolonged mental activities or stress. | A state of exhaustion and decreased cognitive performance, often associated with sustained mental activities or stress. |
| 18 | Motivation/goal setting | The impetus that gives purpose or direction to behavior and operates in humans at a conscious or unconscious level. | The driving force that imbues behavior with purpose or direction, functioning at both conscious and unconscious levels in humans. |
| 19 | Perceived behavioral control | The extent to which an individual believes a behavior is under his/her active control. | The degree to which a person believes they have active control over their behavior. |
| 20 | Enjoyment | The subjective experience characterized by the pleasure and enthusiasm involved in its practice. | A subjective experience characterized by the sense of pleasure and enthusiasm derived from its practice. |
| 21 | Self-efficacy | An individual's subjective perception of their capability to perform in a given setting or to attain desired results. | An individual's subjective perception of their capability to perform in a given setting or to attain desired results. |
| 22 | Parental (role) modeling | A process of observational learning in which the behavior of the parent acts as a stimulus for similar interest or behavior in their child. | A learning process through observation, where the behavior exhibited by the parent serves as a stimulus for similar interest or behavior in their child. |
| 23 | PABs history and patterns | Individual's past engagement in physical activity and the recurring habits observed over time. | A person's historical engagement in physical activity and the consistent habits identified over a given period. |
| 24 | Sedentary behaviour | Any waking behavior characterized by an energy expenditure ≤1.5 metabolic equivalents (METs), while in a sitting, reclining or lying posture. | Any waking behavior characterized by an energy expenditure ≤1.5 metabolic equivalents (METs), while in a sitting, reclining or lying posture. |
| 25 | Phone usage | The frequency and duration a phone is used. | The frequency and duration of mobile phone utilization. |
| 26 | Independent active mobility | Freedom to move around in their local environment without accompaniment. | Freedom to move around in one's local environment without accompaniment. |
| 27 | Sleep | A circadian state characterized by partial or total suspension of consciousness, voluntary muscle inhibition, and relative insensitivity to stimulation. | A circadian state characterized by partial or total suspension of consciousness, voluntary muscle inhibition, and relative insensitivity to stimulation. |
| 28 | Participation in organised sports | The frequency, duration and/or intensity in practising physical activity that involves rules and formal trainining and competition, organised by recognised sport organisations. | The frequency, duration, and/or intensity of physical activity participation that involves predetermined rules, formal training, and competition, coordinated by acknowledged sport organisations. |
| 29 | Active transport | Physical activity undertaken as a means of transport and includes walking, cycling or other non-motorised vehicles. | Physical activity executed as a mode of transportation, encompassing actions such as walking, cycling, or utilizing other non-motorised vehicles. |
| 30 | Backyard access/size | Availability and reachability of a space close to one's livng or working facility (e.g. school, office, nursing home). | Availability and reachability of a space in proximity to one's residential or working location (e.g. school, office, nursing home). |
| 31 | Green space access | Availability and reachability of a space in nature close to one's livng or working facility (e.g. school, office, nursing home). | The accessibility and closeness of a natural environment in relation to a person's residential or occupational location, such as a school, office, or care facility. |
| 32 | PA provision and ethos in setting | The opportunities provided for individuals to be physically active. | The opportunities afforded for individuals to engage in physical activity within a certain environment. |
| 33 | Neighbourhood characteristics | The demographic, social, built environment, or economic features of a geographic area in which people live. | The demographic, social, architectural, or economic attributes of a geographic area where individuals reside. |
| 34 | Provision proximity (parks/playground) | Distance from specific location to parks/playground. | The geographical distance between a specific location and recreational green spaces such as parks or playgrounds. |
| 35 | Access to sport/recreational facilities | Reachability and availability to the closest sport/recreational facility. | The ease of reaching and the availability of the nearest sport or recreational facility. |
| 36 | Time outdoors | Duration of sports and other leisure activities carried out outside buildings. | The duration of engaging in sports and other leisure activities conducted outside of enclosed structures. |
| 37 | Availability of physical activity programs and equipment within schools and community | The presence of and access people have to the physical activity projects and equipment in schools and community. | The existence of and the accessibility to physical activity initiatives and equipment within educational institutions and local community areas. |
| 38 | Cultural perspective on PABs | Historical and social factors influencing individuals’ attitudes, beliefs, motivations and practices regarding physical activity. | The influence of historical and societal factors on individuals' attitudes, beliefs, motivations, and practices concerning physical activity. |
| 39 | Group/family/peer support | Individual’s perception that someone is cared for, esteemed and valued by family, friends, colleagues or others. | A person's perception of receiving care, esteem, and value from their family, friends, colleagues, or others. |
| 40 | Companionship | Engagement in activities with others who share common experiences, interests, values or goals. | The presence and support of a companion or friend, often providing emotional and social support. |
| 41 | Social contact | Interactions with others, which involves face-to-face or media-related activities. | Interactions with others, which involves face-to-face or media-related activities. |
