# Supplementary material for: Definitions of determinants of physical activity behaviour: process and outcome of consensus from the DE-PASS expert group
Source: Int J Behav Nutr Phys Act. 2025 Mar 18;22:34. doi: 10.1186/s12966-025-01728-5 (PMC11921651; doi:10.1186/s12966-025-01728-5)
Supplement: Supplementary file 3 — Additional file 3. Final definitions. [file 12966_2025_1728_MOESM3_ESM.docx]

| Supplementary File 3: Final definitions of determinants | | | |
| --- | --- | --- | --- |
| Determinant | Round 2 | Agreement* (%) | Final definition^1^ |
|  | Original wording |  |  |
| Age | The span of time since a person's birth, generally marked in years or months. Also known as chronological age. | **95.7** | The span of time since an **individual’s** birth, generally marked in years or months. Also **referred to** as chronological age. |
| Sex | Pertains to the biological and physiological distinctions, typically involving variations in reproductive systems, chromosomal patterns, and hormone profiles, most often classified as male or female. | **89.9** | **Biological** and physiological distinctions, typically involving variations in reproductive systems, chromosomal patterns, and hormone profiles, most often classified as male or female. |
| Socioeconomic status | The position of an individual or group in the society, based on a combination of education, income, occupation and social factors. | **94.2** | The position of an individual or group **within** society, based on a combination of education, income, occupation, and **other** social factors. |
| Ethnicity | A shared identity rooted in cultural, historical, religious, and traditional facets derived from a common geographic or national origin, differentiating certain population subgroups from others. | **85.5** | A shared identity **that distinguishes between different subgroups, based on a** common geographical **or** national origin**, and rooted in cultural, historical, religious, and traditional facets.** |
| Education attainment level | The highest level of schooling completed by a person or a group of people. | **98.6** | The highest level of schooling or education completed by an individual **or group**. |
| Setting | Specific environment or context within which particular behaviours are observed. | **92.8** | **A** specific environment or context **in** which particular behaviours are observed. |
| Household income | The aggregate incomes of all members of a household, adjusted for the number of members and overall household size, over a specific time period. | **85.5** | The aggregate **income** of all members of a household over a specific time **period**, adjusted for the number of members and the overall household size. |
| Health Status | The extent to which a person or specified group can engage in and fulfill anticipated roles and functions on physical, mental, emotional, and social levels. | **78.3** | The extent to which **an individual** or group can **perform** or engage in anticipated roles and functions, **at** a physical, mental, emotional, and social level. |
| Stress | A physiological or psychological response to intense physical, mental, or emotional demands or challenges, either from within or external sources. | **87** | A physiological or psychological **reaction** to intense physical, mental, or emotional demands or challenges, **stemming** from internal or external sources. |
| Life events | Social, psychological, and environmental occurrences that require adaptation or trigger a change in an individual's pattern of living. | **87** | Social, psychological, and environmental occurrences that require adaptation or trigger a change in an individual's pattern of living. |
| Physical fitness | Individual's capacity to perform physical activity including components such as cardiorespiratory fitness, musculoskeletal fitness (i.e., muscular endurance and strength), flexibility and body composition. | **85.5** | **An** individual's capacity to **perform** physical **activities**, including components such as cardiorespiratory fitness, musculoskeletal fitness (i.e., muscular endurance and strength), flexibility**,** and body composition. |
| Heart rate | The frequency at which a person's heart contracts within a given time, typically per minute. | **92.8** | The frequency at which **an individual’s** heart contracts within a **specific time period**, typically per minute. |
| Body fat | Specialized tissue, known as adipose, with distinctive metabolic and endocrine functions, often considered in the context of body composition. | **88.4** | Specialised tissue known as adipose, with distinctive metabolic and endocrine functions, **and** often addressed **within** the context of body composition. |
| Genetic profile and regulation | A set of characteristics unique to an individual or group's DNA, utilized for predicting disease susceptibility or diagnosis, understanding disease progression, or assessing responses to pharmaceutical or radiation therapies. | 69.6 | The agreement in round 2 did not achieve the required threshold |
| Perceived competency | A person's conviction about their capacity to perform certain tasks effectively. | **92.8** | **An individual's** conviction **about their ability** to perform **a given** task **in an effective and efficient manner**. |
| Self-regulation | An individual's ability to manage and monitor their emotions, behaviors, and desires amidst external demands in order to function in society. | **84.1** | An individual's ability to manage and monitor their emotions, behaviours, and desires **subject to** external demands, **as a means for functioning within** society. |
| Mental fatigue | A state of exhaustion and decreased cognitive performance, often associated with sustained mental activities or stress. | **91.3** | A state of exhaustion and decreased cognitive performance, often associated with **prolonged** mental activities or stress. |
| Motivation/goal setting | The driving force that imbues behavior with purpose or direction, functioning at both conscious and unconscious levels in humans. | **92.8** | The driving force that **instils** purpose or direction in an individual’s behaviour, at both a conscious and unconscious level. |
| Perceived behavioural control | The degree to which a person believes they have active control over their behavior. | **94.2** | The degree to which **an individual** believes they **have** active control **of** their behaviours. |
| Enjoyment | A subjective experience characterized by the sense of pleasure and enthusiasm derived from its practice. | **92.8** | A subjective experience **that is** characterised by a sense of pleasure and enthusiasm **that is derived** **from that experience**. |
| Self-efficacy | An individual's subjective perception of their capability to perform in a given setting or to attain desired results. | **92.8** | An individual's subjective perception of their capability to perform in a given setting or to attain desired results. |
| Parental (role) modelling | A learning process through observation, where the behavior exhibited by the parent serves as a stimulus for similar interest or behavior in their child. | **92.8** | A learning process through observation, where the behaviour exhibited by the parent serves as a stimulus for similar **interests** or **behaviours** in their child. |
| PABs history and patterns | A person's historical engagement in physical activity and the consistent habits identified over a given period. | **82.6** | An **individual's** historical engagement in physical activity and **their** consistent habits**, as** identified over a **specific time** **period**. |
| Sedentary behavior | Any waking behavior characterized by an energy expenditure ≤1.5 metabolic equivalents (METs), while in a sitting, reclining or lying posture. | **92.8** | Any waking behavior characterized by an energy expenditure ≤1.5 metabolic equivalents (METs), while in a sitting, reclining or lying posture. |
| Phone usage | The frequency and duration of mobile phone utilization. | **84.1** | The frequency and duration of mobile phone **use**. |
| Independent active mobility | Freedom to move around in one's local environment without accompaniment. | **89.9** | **The individual’s ability to physically** move around **within their** environment without **assistance.** |
| Sleep | A circadian state characterized by partial or total suspension of consciousness, voluntary muscle inhibition, and relative insensitivity to stimulation. | **92.8** | A circadian state characterized by partial or total suspension of consciousness, voluntary muscle inhibition, and relative insensitivity to stimulation. |
| Participation in organised sports | The frequency, duration, and/or intensity of physical activity participation that involves predetermined rules, formal training, and competition, coordinated by acknowledged sport organisations. | **89.9** | The frequency, duration**, and** intensity of physical activity **performance that entails predefined** rules, formal training, and competitions**, and that is held by a formal** sports organisation. |
| Active transport | Physical activity executed as a mode of transportation, encompassing actions such as walking, cycling, or utilizing other non-motorised vehicles. | **91.3** | **Performing** physical activity as a mode of transport**, such as** walking, cycling**, and other** non-motorised **means.** |
| Backyard access/size | Availability and reachability of a space in proximity to one's residential or working location (e.g. school, office, nursing home). | 73.9 | The agreement in round 2 did not achieve the required threshold |
| Green space access | The accessibility and closeness of a natural environment in relation to a person's residential or occupational location, such as a school, office, or care facility. | **89.9** | The accessibility and closeness of a natural environment in relation **to the individual’s place of residence or work (e.g., a school, office, or care facility).** |
| Physical activity provision and ethos in setting | The opportunities afforded for individuals to engage in physical activity within a certain environment. | **82.6** | **The opportunities available to the individual for engaging in physical activity within a given environment.** |
| Neighbourhood characteristics | The demographic, social, architectural, or economic attributes of a geographic area where individuals reside. | **89.9** | The demographic, social, architectural, or economic attributes of a geographic area **in which** individuals reside. |
| Provision proximity (parks/playground) | The geographical distance between a specific location and recreational green spaces such as parks or playgrounds. | **92.8** | The geographical distance between a **given** location and recreational green spaces **(e.g., parks or playgrounds).** |
| Access to sports/recreational facilities | The ease of reaching and the availability of the nearest sport or recreational facility. | **87** | **The accessibility and closeness of the nearest sports or recreational facility** to the individual’s home or work**.** |
| Time outdoors | The duration of engaging in sports and other leisure activities conducted outside of enclosed structures. | **82.6** | The duration **spent** engaging in sports and other leisure **activities outside** of enclosed structures. |
| Availability of physical activity programs and equipment within schools and the community | The existence of and the accessibility to physical activity initiatives and equipment within educational institutions and local community areas. | **84.1** | The existence **and accessibility of** physical activity initiatives and equipment within educational institutions and local community **settings**. |
| Cultural perspective on PABs | The influence of historical and societal factors on individuals' attitudes, beliefs, motivations, and practices concerning physical activity. | **87** | The **impact** of historical and societal factors on **the individual’s** attitudes, beliefs, motivations, and practices **regarding** physical activity. |
| Group/family/peer support | A person's perception of receiving care, esteem, and value from their family, friends, colleagues, or others. | **91.3** | An **individual's** **perceptions** of receiving care, **encouragement**, and value from their family, friends, colleagues, or others. |
| Companionship | The presence and support of a companion or friend, often providing emotional and social support. | **85.5** | The **physical** presence of a companion or friend**, who tends to provide** emotional and social support**.** |
| Social contact | Interactions with others, which involves face-to-face or media-related activities. | **89.9** | Interactions with others, which **involve** face-to-face or media-related activities. |
| *Percentage of experts reporting “Strongly agree” or “Somewhat Agree” in the modified Delphi round 2.  ^1^Final version of definition resulted from Final consensus of the Steering Committee.  Note: Bold % corresponds to the achievement of the ≥75% threshold for agreement; bold text corresponds to changes made from the original version. | | | |
